# SPICE-Compatible Degradation Modeling Framework for TDDB and LER Effects in Advanced Packaging BEOL Based on Ion Migration Mechanism

**DOI:** 10.3390/mi16070766

**Published:** 2025-06-29

**Authors:** Shao-Chun Zhang, Sen-Sen Li, Ying Ji, Ning Yang, Yuan-Hao Shan, Li Hong, Hao-Gang Wang, Wen-Sheng Zhao, Da-Wei Wang

**Affiliations:** 1 Information Science Academy, China Electronics Technology Group Corporation, Beijing 100142, China; zhangscchn@163.com (S.-C.Z.); yangning8848@163.com (N.Y.); shanyuanhao_uestc@163.com (Y.-H.S.); hongli1993@163.com (L.H.); 2 National Key Laboratory of Integrated Circuits and Microsystems, Beijing 100142, China; 3 Innovation Center for Electronic Design Automation Technology, Hangzhou Dianzi University, Hangzhou 310018, China; 232040123@hdu.edu.cn (S.-S.L.); wshzhao@hdu.edu.cn (W.-S.Z.); 4 College of Information Science and Electronic Engineering, Zhejiang University, Hangzhou 310027, China; hgwang@zju.edu.cn

**Keywords:** time-dependent dielectric breakdown, packaging dielectric layer, SPICE-compatible TDDB model, LER-SPICE model

## Abstract

The time-dependent dielectric breakdown (TDDB) degradation mechanism, governed by the synergistic interaction of multiphysics fields, plays a pivotal role in the performance degradation and eventual failure of semiconductor devices and advanced packaging back-end-of-line (BEOL) structures. This work specifically focuses on the dielectric breakdown mechanism driven by metal ion migration within inter-metal dielectric layers, a primary contributor to TDDB degradation. A SPICE-compatible modeling approach is developed to accurately capture the dynamics of this ion migration-induced degradation. The proposed model is rooted in the fundamental physics of metal ion migration and the evolution of conductive filaments (CFs) within the dielectric layer under operational stress conditions. By precisely characterizing the degradation behavior induced by TDDB, a SPICE-compatible degradation model is developed. This model facilitates accurate predictions of resistance changes across a range of operational conditions and lifetime, encompassing variations in stress voltages, temperatures, and structural parameters. The predictive capability and accuracy of the model are validated by comparing its calculated results with numerical ones, thereby confirming its applicability. Furthermore, building upon the established degradation model, the impact of line-edge roughness (LER) is incorporated through a process variation model based on the power spectral density (PSD) function. This PSD-derived model provides a quantitative characterization of LER-induced fluctuations in critical device dimensions, enabling a more realistic representation of process-related variability. By integrating this stochastic variability model into the degradation framework, the resulting lifetime prediction model effectively captures reliability variations arising from real-world fabrication non-uniformities. Validation against simulation data demonstrates that the inclusion of LER effects significantly improves the accuracy of predicted lifetime curves, yielding closer alignment with observed device behavior under accelerated stress conditions.

## 1. Introduction

As electronic packaging continues to advance toward higher performance and greater integration density, the ongoing miniaturization of chip dimensions and the widespread adoption of advanced packaging BEOL technologies have enabled the vertical stacking of multiple dies. This paradigm shift effectively reduces signal propagation delays and significantly enhances system-level integration and overall performance. However, the increasing number of stacked chips, coupled with reduced feature sizes and elevated packaging densities, introduces a host of new reliability challenges. These include thermal management issues, electromigration, thermomigration, time-dependent dielectric breakdown (TDDB), and various interacting degradation mechanisms [1,2,3]. Among these, TDDB has emerged as a particularly critical reliability concern. This is primarily due to the narrowing spacing between metal interconnects, increased electric field strengths, and elevated operating temperatures—factors that synergistically accelerate the onset and progression of TDDB failure mechanisms [4,5,6,7]. TDDB not only undermines device longevity and operational stability but can also precipitate catastrophic system-level failures. Consequently, the accurate characterization, modeling, and analysis of TDDB-induced degradation in both devices and interconnect structures—as well as the underlying failure mechanisms—have become essential and increasingly complex focal points in the field of integrated microsystem reliability.

TDDB refers to a progressive degradation mechanism wherein prolonged electrical stress induces the gradual migration of atoms within the dielectric layer, ultimately leading to the formation of conductive paths. This process results in a gradual increase in leakage current, culminating in catastrophic dielectric failure via electrical breakdown. In the initial stages of TDDB, the variation in leakage current remains relatively modest. However, as the duration and intensity of electrical stress increase, the leakage current begins to rise at an accelerating rate, eventually exhibiting a sharp exponential surge that signifies imminent dielectric failure. Under higher electric fields, dielectric materials exhibit significantly reduced electrical stability, which promotes the rapid nucleation and growth of conductive filaments (CFs). Consequently, both the magnitude and the rate of change of the leakage current increase substantially [8,9]. This behavior imposes stringent reliability requirements on the selection and design of dielectric materials used in semiconductor integrated circuits (ICs) [10]. Therefore, it is crucial to conduct in-depth investigations into the behavioral characteristics of TDDB under diverse operational conditions.

Traditionally, TDDB characterization has primarily focused on gate oxides in transistors. In response to the complex nature of TDDB under various stress conditions, numerous empirical and physics-based lifetime models have been proposed. These include the E model, 1/E model, E^1/2^ model, power-law model, and fatigue damage model [11,12,13,14]. In [15], Shihan Zhao investigated the creep–fatigue interaction failure mechanism of solder layers under power cycling test (PCT) conditions. An ultra-high-resolution computed tomography (CT) scanner was employed to continuously monitor the microstructural evolution of the solder layer during the degradation process. The results demonstrated that even when the thermal resistance remained constant, significant changes occurred in the microstructure of the solder layer. A. Vici introduced a multi-level-independent defect generation framework to describe the evolution of defects in gate oxides under TDDB stress. This approach assumes that defect generation within the dielectric is proportional to both the energy and the quantity of injected charge carriers [16]. Building upon this foundation, Vici further developed an analytical Markov-based model capable of predicting TDDB failure times in metal–oxide–semiconductor (MOS) systems under arbitrary voltage and temperature stress conditions [17], thereby enabling accurate reliability estimation across a wide range of operational scenarios. In contrast, S. Peng proposed an electron pathway generation model tailored for intra-layer dielectrics in copper interconnects, where defect formation arises from the diffusion of metal ions within the dielectric matrix [18]. However, as device dimensions continue to scale down, process variations increasingly influence structural integrity and reliability. For instance, in [19], a novel comprehensive modeling approach was proposed to assess the impact of line-edge roughness (LER) on TDDB behavior. Their findings revealed that LER not only significantly reduces the scale parameter of the Weibull distribution but also decreases the shape parameter, indicating a broader variability in time-to-failure distributions. Additionally, studies in [20,21] presented a time-to-failure model incorporating LER effects for predicting TDDB reliability in scaled copper interconnects. The validity and accuracy of the model were rigorously evaluated, demonstrating its applicability across conventional and spacer-defined nanoscale patterning techniques. These results underscore the criticality of accounting for LER-induced variability in reliability assessments. Despite these advancements in modeling dielectric degradation within devices and packaging structures, there remains a notable gap in the development of circuit-level TDDB degradation models that incorporate LER effects in advanced interconnect architectures. Hence, it is essential to establish a SPICE-compatible lifetime prediction model that integrates LER-induced variability, enabling a more holistic and realistic reliability evaluation of modern electronic systems.

This study develops a physics-based, SPICE-compatible degradation model for inter-metal dielectric layers, capturing both breakdown (BD)-induced failure and LER-driven resistance degradation in advanced packaging interconnects. The remainder of the paper is structured as follows. Section 2 provides an overview of the relevant research background and simulation methodologies associated with the electron pathway generation (EPG) model. Section 3 presents a comprehensive description of the proposed modeling framework, including the development of the SPICE degradation model, the LER-based process variation model, and their integration into the overall simulation environment. Section 4 offers an in-depth analysis and discussion of the simulation results, highlighting the impact of TDDB and LER on circuit performance degradation. Finally, Section 5 concludes the paper with a summary of key findings and potential directions for future work.

## 2. Review of EPG Model for TDDB

**Figure 1:**
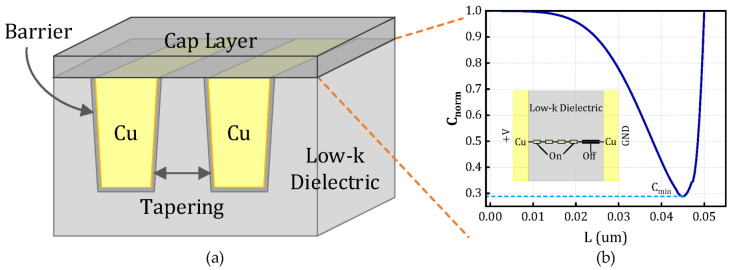
(**a**) Schematic diagram of advanced interconnect with low-κ substrate and the related TDDB process and (**b**) distribution of normalized ion concentration in the dielectric layer at various distances from the anode (Cₘᵢₙ: the minimum ion concentration within the intermetal layer).

This section begins with an overview of the EPG model for time-dependent dielectric breakdown (TDDB), as proposed in [18]. As illustrated in Figure 1, this model focuses on the metal–insulator–metal (MIM) structure and analytically examines the time evolution of metal ion concentration within the dielectric layer. By introducing a concentration threshold criterion, the model enables the prediction of the dielectric time-to-failure (TTF).

Based on the insights obtained from the EPG model, barrier metal ions are injected into the dielectric layer under electrical stress. In this study, particular attention is given to the two-dimensional diffusion behavior of these ions at the interface between the inter-metal dielectric (IMD) and the oppositely charged metal electrodes. The simulation results indicate that TDDB-induced failure predominantly initiates within this interfacial region.

In the proposed framework, the local electrical conductivity is assumed to be proportional to the hopping probability of electrons between adjacent defect centers. As metal ion diffusion progresses, defects are generated within the dielectric matrix, acting as potential sites for electron localization originating from the metal electrodes. These interconnected defect centers collectively form a distributed resistive network. The resistance between any two defect centers i and j is determined according to Equations (1) and (2), which govern the electron transport characteristics within the evolving conductive pathways.

(1) $$R_{\mathrm{ij}}=R_{\mathrm{ij}}^{0}\;\exp\;\left\{-\frac{2r_{\mathrm{ij}}}{a}-\frac{\varepsilon_{\mathrm{ij}}}{k_{B}T}\right\}$$

(2) $$r_{ij}=C\left(x,y,t\right)-1/2$$

where $r_{\mathrm{ij}}$ is the distance between i and j centers, a is the radius of electron localization at this type of centers (analog of Bohr’s radius), which can reach 100 Å, $\varepsilon_{\mathrm{ij}}$ is the energy barrier between centers, $k_{B}$ is the Boltzmann constant, T is the absolute temperature, $k_{B}T$ is the thermal energy, and C(x,y,t) is the ion concentration at the considered interface as a function of spatial coordinates and time.

Figure 1 illustrates the corresponding resistive network within the IMD at an arbitrary time, along with the ion concentration distribution along the conduction path from point (0, d) connecting the metal electrodes. According to Equations (1) and (2), a higher ion concentration corresponds to a lower resistance. When the total resistance along the (0, d) path decreases to a predefined threshold, the formation of an electrical conduction path is considered to occur, indicating the onset of TDDB failure. Due to the exponential dependence of resistance on the distance between adjacent conductive centers, the total resistance along the (0, d) path is predominantly determined by the largest individual resistance—that is, the location with the lowest ion concentration in the dielectric layer. Consequently, the threshold resistance of the conduction path can be reasonably approximated as being governed by the minimum ion concentration. The normalized ion concentration distribution, defined as $C_{\mathrm{norm}}\left(x,\;y,\;t\right)=C(x,\;y,\;t)/C_{0}$, is governed by the ion transport equation under an applied electric field, as described by Equations (3) and (4).

(3) $$\frac{\partial C_{\mathrm{norm}}}{\partial t}=-\nabla J,\;\mathrm{where}\;J=-D\;\nabla C_{\mathrm{norm}}+v_{d}C_{\mathrm{norm}}$$

The boundary conditions are given by

(4) $$C_{\mathrm{norm}}(x=0)=C_{\mathrm{norm}}(x=d)=1$$

where J is the metal ion flux, $D=D_{0}\exp(-E_{a}/k_{B}\cdot T)$ is the diffusion coefficient, $v_{d}=E(q\cdot D/k_{B}\cdot T)$ is the drift velocity of metal ions, $E_{a}$ is the activation energy, q is the elementary charge, and E is the electric field. The distance d is considered to be the minimum spacing between two metal lines within the IMD.

In the one-dimensional case, the governing drift-diffusion equation, along with the corresponding boundary and initial conditions, can be expressed as in Equations (5)–(7).

(5) $$D\;\frac{\partial^{2}C}{\partial x^{2}}=\frac{q\;D\;E}{k_{B}\;T}\cdot \frac{\partial C}{\partial x}+\frac{\partial C}{\partial t}$$

The boundary conditions are given by

(6) C(Anode and Cathode)=1

The initial condition is defined as

(7) C(t=0, Within the Dielectric)=0

## 3. TDDB SPICE Modeling Method

In this section, a macroscopic characterization of the TDDB mechanism in the dielectric layer is presented, based on the physical evolution of CF formation. Accordingly, a SPICE-compatible degradation model is proposed to enable a circuit-level simulation and reliability assessment of TDDB effects under various operational conditions. Further, a LER-based process variation model is introduced to account for manufacturing-induced dimensional and structural fluctuations in interconnect geometries. Finally, an integrated modeling and simulation framework is established, incorporating LER-aware effects into the TDDB degradation model. This framework enables comprehensive characterization of dielectric failure mechanisms at both device and circuit levels, facilitating more accurate reliability predictions for advanced electronic packaging technologies.

### 3.1. CF Compact Model

In the inter-metal dielectric layer, atoms gradually accumulate along preferential diffusion paths due to electrostatic forces, eventually forming a narrow conductive filament (CF) that bridges the two electrodes. Once the CF is fully formed, the dielectric undergoes a significant increase in electrical conductivity, leading to a sharp decline in its insulating capability. This transition initiates a cascade of electrochemical and thermochemical reactions within the dielectric layer. For instance, under certain conditions, the CF may induce device thermal expansion or fracture, increased signal transmission loss, and delay, all of which adversely affect the stability and reliability of the dielectric interface.

**Figure 2:**
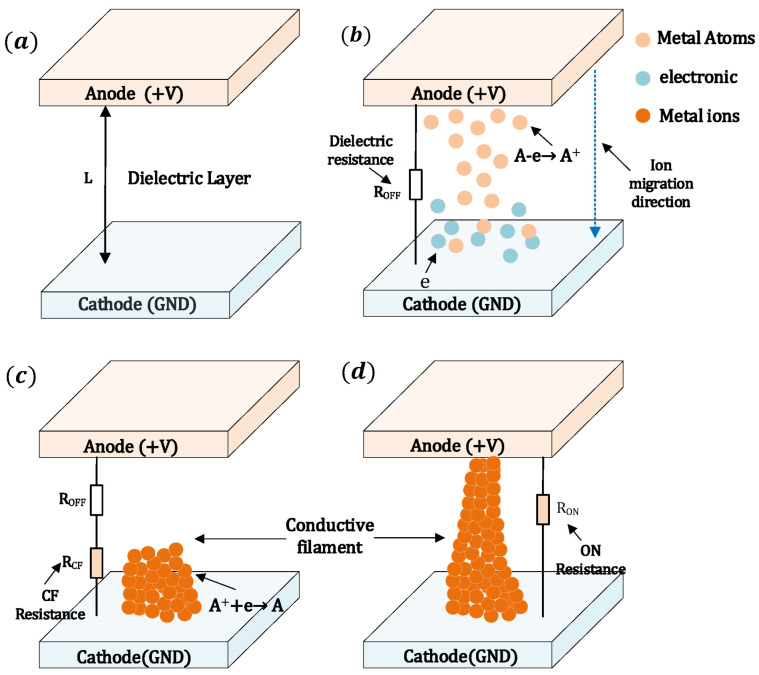
Schematic illustration of the CF formation process in the dielectric layer: (**a**) initial state with no ion migration, (**b**) ion migration from the anode to the cathode through the dielectric, (**c**) filament formation via ion reduction and accumulation, and (**d**) fully formed CF electrodes.

The formation process of the CF is illustrated in Figure 2 [22]. Figure 2a illustrates the initial off-state, where no defects are generated within the dielectric layer and atoms do not undergo migration. Upon applying a forward bias to the active electrode, electrochemical reactions occur at the interface between the active electrode and the dielectric, resulting in atom A losing an electron $A\;-\;e\to \;A^{+}$, as shown in Figure 2b. Subsequently, the $A^{+}$ migrates under the influence of the electric field toward the inert electrode, where it captures an electron near the inert electrode, forming $A^{+}+e\to \;A$. These A atoms gradually accumulate within the dielectric layer, initially forming a narrow CF in the longitudinal direction, followed by lateral growth as depicted in Figure 2c. Eventually, a tapered CF connecting the top and bottom electrodes is formed, as shown in Figure 2d.

**Figure 3:**
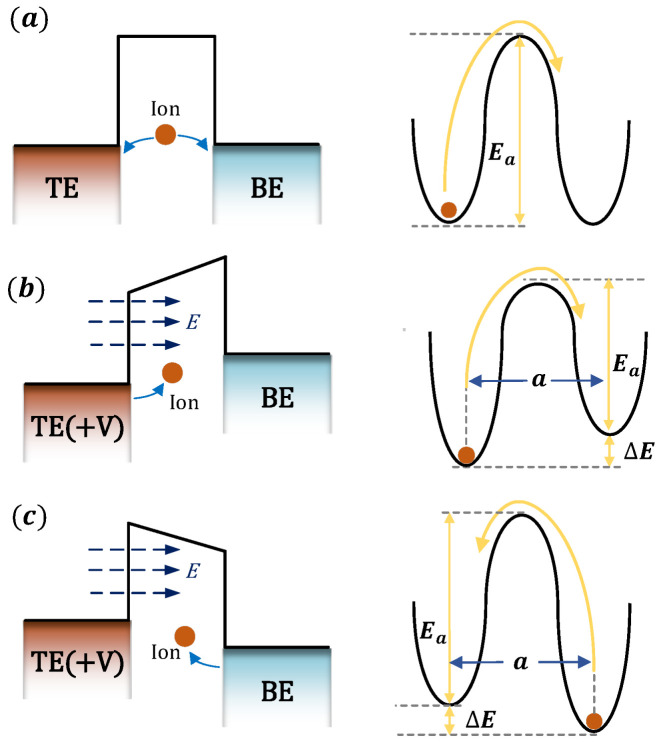
The definition of ion activation energy and migration barriers. (**a**) No external electric field, (**b**) migration towards BE under an external electric field, and (**c**) migration towards TE under an external electric field. a represents the effective hopping distance.

At the macroscopic level, the formation of the CF can be described as the drift of ions and accumulation of atoms [23,24,25]. As shown in Figure 3a, in the absence of an external electric field, the initial migration energy barrier for ions is $E_{a}$. Under this condition, ion migration is random, with equal probabilities of moving left or right, both given by $f\cdot \exp(-E_{a}/(k_{B}T))$. Once an external voltage is applied to the active electrode (top electrode, TE), the migration energy barrier changes: the barrier height for ion migration toward the inert electrode (bottom electrode, BE) decreases to $E_{a}-E$, as shown in Figure 3b, while the barrier height for migration toward the TE increases to $E_{a}+-E$, as shown in Figure 3c. In the presence of an external electric field E, work is performed on charged ions, altering their potential energy. The change in potential energy for ions moving along the direction of the electric field is$\;\mathrm{Zq}E_{a}/2$, while for those moving in the opposite direction, it is$\;-\mathrm{Zq}E_{a}/2$. Consequently, under the influence of the external electric field, the migration energy barriers for ions moving to the left and right become $E_{a}+\mathrm{Zq}E_{a}/2$ and $E_{a}-\;\mathrm{Zq}E_{a}/2$. Under these conditions, the ion migration rates to the left and right can be expressed as

(8) $$P_{\mathrm{left}}=f\;\times \;\exp\left(-\frac{E_{a}+\frac{\mathrm{BZqEa}}{2}}{k_{B}T}\right)$$

(9) $$P_{\mathrm{right}}=f\;\times \;\exp\left(-\frac{E_{a}-\frac{\mathrm{BZqEa}}{2}}{k_{B}T}\right)$$

where f is the attempt frequency, B is the field acceleration factor, and Z is the ion charge number. The drift velocity of the ions$\;\upsilon_{\mathrm{dr}}$ is then calculated as

(10) $$v_{\mathrm{dr}}=\left(\begin{array}{c} P_{\mathrm{right}}-P_{\mathrm{left}} \end{array}\right)\;\times \;a$$

where V is the applied voltage, $V_{0}=2Lk_{B}T/\mathrm{Zqa}$.

Based on the ion drift velocity, the ion drift flux $J_{\mathrm{dr}}$ can be calculated as

(11) $$J_{\mathrm{dr}}=\upsilon_{\mathrm{dr}}\cdot n$$

The formation of the CF within the dielectric is primarily attributed to ion drift and atomic accumulation. Given a time interval dt, the change in CF length is denoted as dh. During this interval, the number of ions drifting through a cross-sectional area S and contributing to CF growth can be calculated based on the increase in the number of atoms within a volume S·dh. Accordingly, the longitudinal growth of the CF can be described by

(12) $$\chi {\;J}_{\mathrm{dr}}\;\left(\frac{\pi \phi^{2}}{4}\right)\;\mathrm{dt}=n\;\left(\frac{\pi \phi^{2}}{4}\right)\;\mathrm{dh}$$

where t is the time, χ is the longitudinal displacement of ions, n is the atomic density, and h and ϕ correspond to the length and diameter of the CF, respectively.

Within a given time interval dt, the change in the base diameter of the CF is denoted as dϕ, resulting in a corresponding volume change in π(ϕ+dϕ)(dϕ/2)h, Accordingly, the lateral growth of the CF diameter can be expressed as

(13) $$\left(1-\chi \right)\;J_{\mathrm{dr}}\;\left(\phi +d\phi \right)\;h\;dt=n\pi \;\left(\phi +d\phi \right)\;\left(\frac{d\phi }{2}\right)\;h$$

As atoms continuously accumulate within the dielectric layer, its electrical properties progressively degrade, ultimately compromising device reliability. Upon the CF bridging the top and bottom electrodes, the dielectric transitions into a low-resistance state. This facilitates a substantial current flow through the dielectric, potentially inducing electrical breakdown and further exacerbating reliability concerns. By jointly solving Equations (10)–(13), the temporal evolution of the CF’s vertical length $h_{E}\;$and lateral diameter $\phi_{E\;}$ can be quantitatively described as

(14) $$\frac{dh_{E}}{\mathrm{dt}}=2\chi \mathrm{fa}\;\exp\;\left(-\frac{E_{a}}{k_{B}\cdot T}\right)\;\sin h\left(\frac{B\cdot V}{V_{0}}\right)$$

(15) $$\frac{d\phi_{E}}{\mathrm{dt}}=\left(\begin{array}{c} \frac{4\left(1-\chi \right)}{\pi } \end{array}\right)\;\mathrm{fa}\;\exp\left(\begin{array}{c} -\frac{E_{a}}{k_{B}\cdot T} \end{array}\right)\;\sin h\left(\begin{array}{c} \frac{B\cdot V}{V_{0}} \end{array}\right)$$

In Equations (14) and (15), the value of V/V_0_ is much smaller than 1, so the sinh function will not cause numerical instability in transient simulations.

### 3.2. TDDB SPICE Model

According to the compact model described, the resistivity of the dielectric layer gradually decreases with the accumulation of atoms [25]. The failure time of the dielectric layer is defined as the moment when the CF penetrates through it. Initially, there is no atomic accumulation within the dielectric layer, and its initial resistance can be calculated as

(16) $$R_{\mathrm{OFF}}=\frac{\rho_{\mathrm{OFF}}L}{S}$$

where $\rho_{\mathrm{OFF}}$ is the resistivity of the dielectric layer, L is the thickness of the dielectric, and $S=\pi /4\cdot {\left[\int_{0}^{t}\left(d\phi_{E})/\mathrm{dt}\right)\;\mathrm{dt}\right]}^{2}$ is the bottom area of the CF. In this initial state, the dielectric layer exhibits excellent insulating properties, low dielectric loss, and favorable transmission characteristics. Under the influence of physical fields such as temperature and electric field, defects gradually emerge within the dielectric layer, leading to atomic migration. As atoms continue to accumulate, the size of the CF increases progressively, while the resistance, insulation, and other characteristics of the dielectric layer begin to deteriorate. During this process, the resistance of the dielectric layer becomes determined by the size of the CF and can be expressed as

(17) $$R_{\mathrm{CF}}=\frac{\rho_{\mathrm{ON}}h+\rho_{\mathrm{OFF}}\left(L\;-\;h\right)}{S}$$

where $\rho_{\mathrm{ON}}$ is the resistivity of the dielectric layer after the formation of the CF, and $h=\int_{0}^{t}\left(dh_{E})/\mathrm{dt}\right)\;\mathrm{dt}$ is the length of the CF.

### 3.3. Process Variation Model

**Figure 4:**
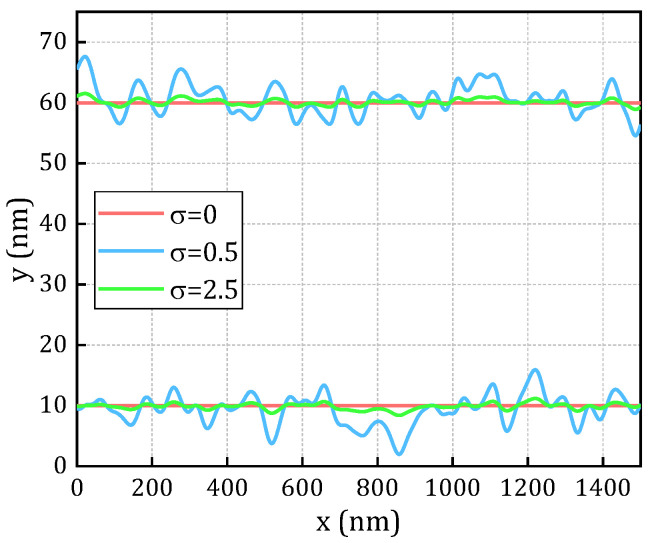
LER profile generated from Gaussian autocorrelation function.

LER and line-to-line spacing (L2L) variations are critical factors affecting the performance of nanoscale devices and interconnects, particularly leading to significant degradation in TDDB performance and reliability. To effectively quantify and evaluate the impact of LER on TDDB effect, a Fourier synthesis technique is developed based on the PSD derived from a Gaussian autocorrelation function (ACF), as reported in [26], to simulate and generate LER profiles. In this approach, the material structure is subdivided into N discrete microcells, and a random phase is assigned to each cell to ensure the stochastic nature and realistic matching of the simulation. The amplitude of the LER generated by the random phase is constrained by the PSD of the Gaussian ACF, thereby ensuring compliance with the statistically observed distribution characteristics from experiments. Specifically, this method characterizes the LER features by

(18) $$S_{G}\left(k\right)=\sqrt{\pi \lambda \sigma^{2}\exp}\;[-(\frac{\lambda^{2}k^{2}}{4})]$$

where $S_{G}$ is the PSD of the Gaussian ACF, and λ and σ represent the correlation length and root mean square (RMS) amplitude of the LER profile, respectively. The wave vector k is defined as k=i(2π/Ndx). dx is the spacing between two adjacent segments, which should be much smaller than λ to ensure adequate discretization resolution. In this study, dx = 0.5, which is significantly smaller than λ, typically ranging from 20 to 50 nm [27], with λ = 30 nm. Figure 4 illustrates the Gaussian ACF and randomly generated LER profiles for a dielectric layer length of 50 nm and RMS amplitudes σ of 0, 0.5, and 2.5 nm, respectively.

### 3.4. TDDB SPICE Modeling Framework

**Figure 5:**
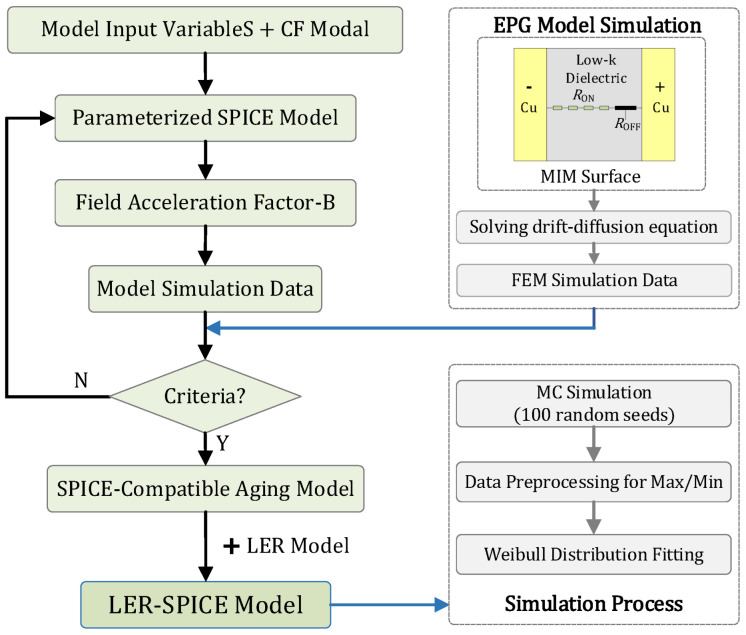
Schematic diagram of the proposed TDDB SPICE modeling framework.

The overall framework of the proposed TDDB SPICE modeling approach is illustrated in Figure 5. The CF model is first combined with the required input parameters to establish a compact representation of the CF evolution process, based on Equations (14)–(17). The EPG model is then employed to simulate the TDDB failure mechanism in the dielectric layer in the BEOL metal interconnect structure, capture the evolution of metal ion concentration within the dielectric, and thereby evaluate and calibrate the proposed degradation model. Following the methodology described in this section, a parameterized SPICE model is developed to describe the TDDB behavior of the MIM structure. Using simulation data obtained from the EPG model, the field acceleration factor B is extracted by fitting, and a SPICE-based degradation model is constructed to characterize the temporal evolution of current and resistance, as well as to enable lifetime prediction. To validate the model’s applicability under varying operational and process conditions, comparisons were conducted by adjusting key model parameters (such as activation energy $E_{a}$ and dielectric layer thickness L), voltage excitation conditions (rectangular and sinusoidal waveforms), and simulations based on the EPG model. Moreover, to assess the impact of process variations on device reliability, a process tolerance model was integrated into the SPICE degradation model, followed by simulation analyses under different stress amplitudes. During this procedure, to ensure the reliability of the simulation data, Monte Carlo simulations were performed using 100 random seeds generated according to the power spectral density function, yielding 100 sets of simulation data. Subsequently, data processing involved excluding outliers by removing the top and bottom 10 datasets. Finally, the Weibull distribution model was applied for fitting and analysis to characterize the failure behavior. Under different stress conditions, the characteristic failure time (t_BD,63%_) was extracted from the fitting results and employed as a critical reference metric for device reliability evaluation.

### 3.5. SPICE Circuit Implementation of TDDB Degradation

**Figure 6:**
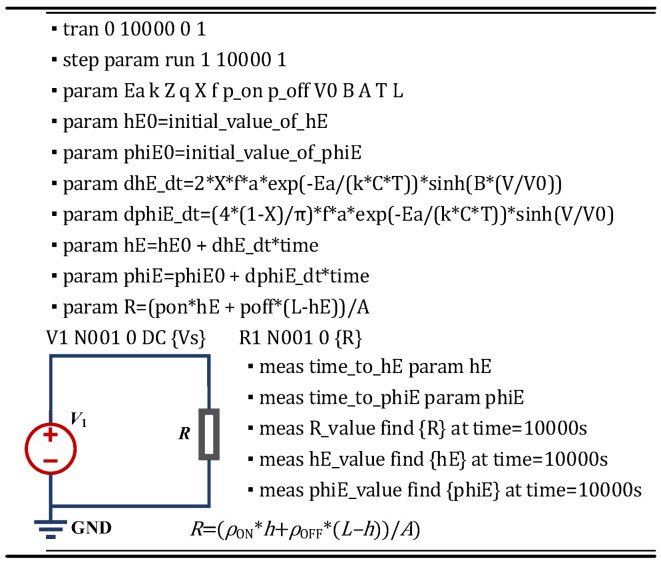
Script and complete expression description of TDDB SPICE Model.

**Figure 7:**
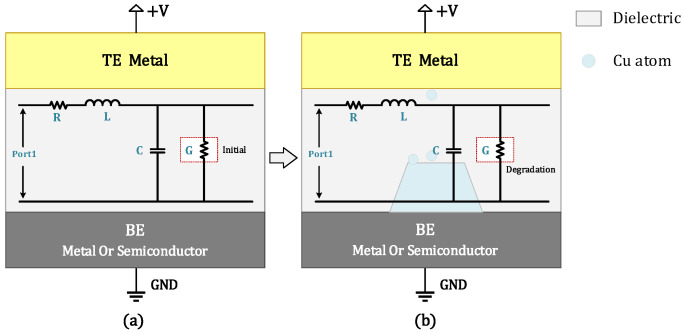
Circuit modeling and SPICE mapping framework for time-dependent TDDB degradation behavior. (**a**) MIM structure and equivalent circuit diagrams at initial state and (**b**) MIM structure and equivalent diagrams after long-term operation (degradation of conductance G).

Based on the mathematical physics equations given in (14) to (15), the TDDB SPICE model can be obtained and represented using the proposed circuit schematic in Figure 6. In Figure 7a, the schematics of the metal–insulator–metal (MIM) structure and its equivalent circuit representing the initial state of the dielectric layer without any degradation are illustrated. Then, with long-term external field stress applied, a conductive metallic filament will gradually form within the dielectric layer due to the migration of ions, causing changes in both the material properties and geometric morphology of the dielectric. With the formation of metal filaments, the conductivity of the dielectric layer usually increases gradually. The corresponding degradation process and equivalent circuit diagram are shown in Figure 7b. Accordingly, a circuit model that can characterize the performance degradation behavior of the dielectric layer under external stress can be constructed. Numerous studies have demonstrated that structural variations during this process lead to significant degradation of circuit performance parameters [28,29].

## 4. Results and Discussions

This section begins with an analysis of the TTF simulation results obtained from the EPG model. Based on these results, a field-acceleration-factor-based SPICE-compatible degradation model is developed, providing a quantitative framework for predicting device degradation under various stress conditions. Building upon this foundation, a process tolerance model is incorporated into the SPICE degradation framework to account for manufacturing-induced variability. A total of 100 independent datasets are generated through Monte Carlo simulations, each representing a statistically significant variation in process parameters derived from the PSD function. The failure behavior of these datasets is characterized using the Weibull distribution model, ultimately yielding the characteristic failure time t_BD,63%._

### 4.1. Simulation Results of the EPG Model

**Figure 8:**
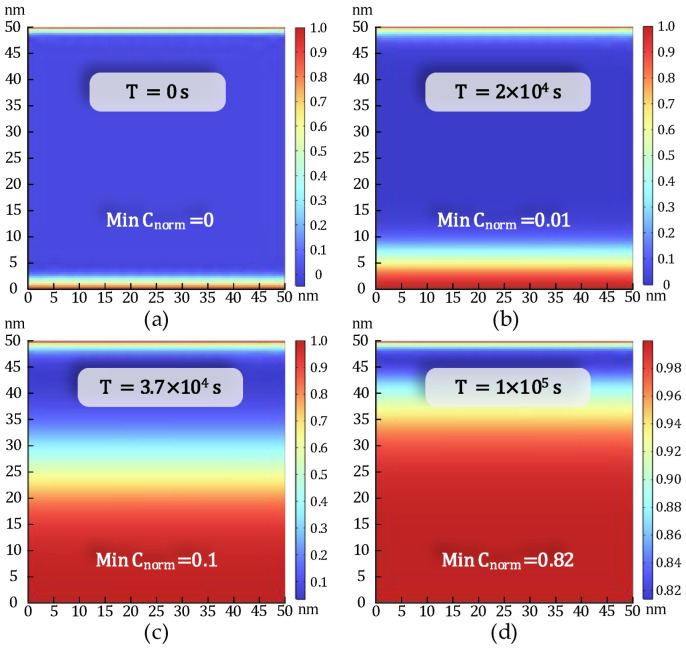
Normalized ion concentration distribution in the dielectric layer at different times in the MIM structure at 0.5 V and 400 K. (**a**) T = 0 s, Min C_norm_ = 0, (**b**) T = 2 × 10^4^ s, Min C_norm_ = 0.01, (**c**) T = 3.7 × 10^4^ s, Min C_norm_ = 0.1, and (**d**) T = 1 × 10^5^ s, Min C_norm_ = 0.82.

**Figure 9:**
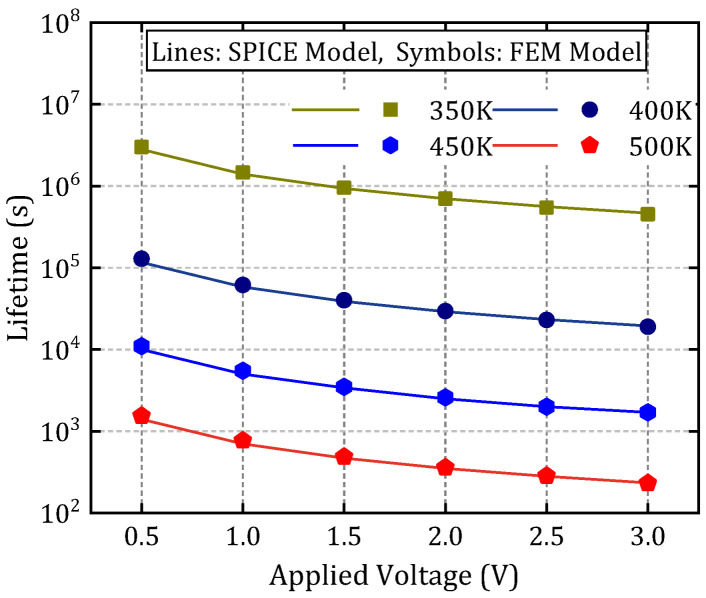
Comparison between finite element simulation and SPICE model-based lifetime prediction.

Based on the EPG model outlined in Section 2, a normalized minimum ion concentration threshold of $C_{norm}=0.95$ is defined along the path (0, d) to serve as the failure criterion for dielectric breakdown [30]. As illustrated in Figure 1, one terminal of the structure is connected to the supply voltage $V_{DD}$, while the other is grounded (GND), with the dielectric material occupying the intervening region. Finite element simulations are performed using the structural configuration shown in Figure 1 and the parameter set detailed in Table 1, aimed at solving the drift-diffusion equation governing ion transport under electrical and thermal stress conditions. The temporal evolution of $C_{\mathrm{norm}}$ within the MIM structure is depicted in Figure 8, demonstrating the progressive accumulation of mobile ions leading to conductive filament formation. Figure 9 presents a comparison between finite element simulation results and SPICE-based lifetime predictions under varying temperature and voltage bias conditions, highlighting the dependence of TDDB degradation on applied stress factors.

### 4.2. Validation of the SPICE Degradation Model

**Figure 10:**
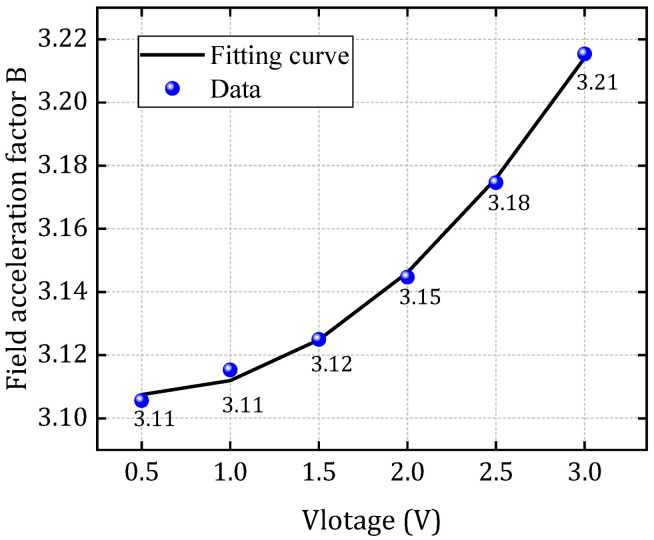
The fitted curve of field acceleration factor as a function of voltage.

Using the structure depicted in Figure 1 and the parameter values summarized in Table 2, a SPICE-compatible degradation model is established based on the theoretical foundation of the EPG model. TTF data obtained from finite element simulations under conditions of V = 1 V and T = 370 K is utilized as training samples for model calibration. The expression of the field acceleration factor as a function of voltage is obtained through curve fitting as B = 0.0169∙V^2^ − 0.0164∙V + 3.11, and the comparison between the original data and fitted data is illustrated in Figure 10, with a relative error smaller than 1%. To validate the model’s predictive capability across different operating conditions, the resistance degradation behavior predicted by the SPICE model is compared with FEM simulation results under varying voltage and temperature levels. The validation is performed over a temperature range of 350 K to 450 K and a voltage range of 0.5 V to 3 V. The relative errors between the calculation results of the SPICE model and the numerical model are summarized in Table 3, in which all relative errors remain below 1%, thereby confirming the model’s accuracy and robustness across a wide range of stress conditions.

**Figure 11:**
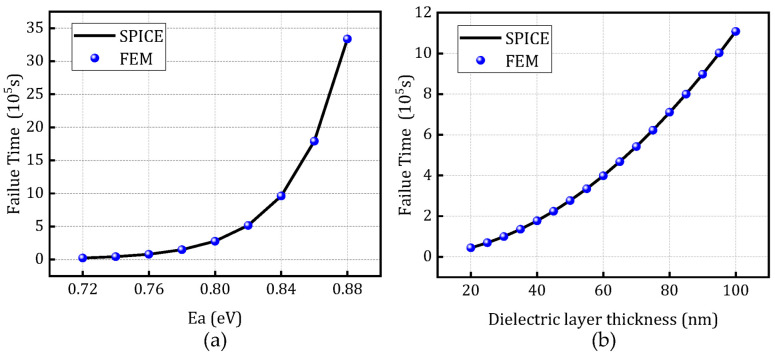
Comparison of time-to-failure between the SPICE degradation model and fem: (**a**) different activation energies and (**b**) dielectric layer thickness.

**Figure 12:**
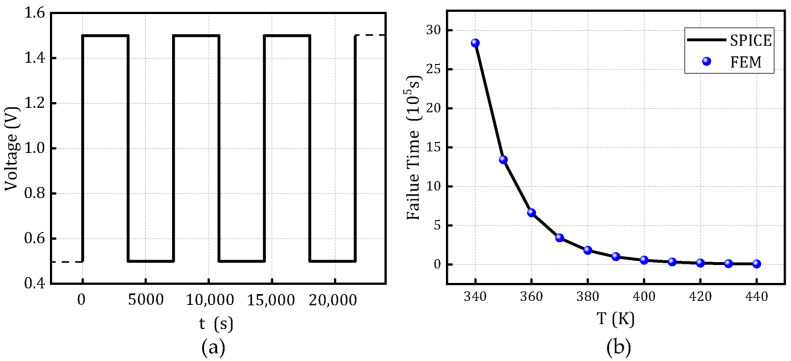
Time-to-failure distribution of the SPICE degradation model and FEM under rectangular voltage waveforms at different temperatures. (**a**) rectangular voltage and (**b**) Failure time at different temperatures.

**Figure 13:**
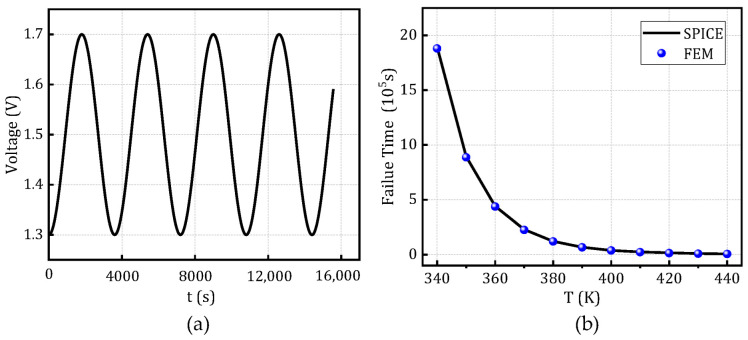
Time-to-failure distribution of the SPICE degradation model and FEM under sinusoidal voltage waveforms at different temperatures. (**a**) sinusoidal voltage and (**b**) Failure time at different temperatures.

To validate the scalability of the constructed model, Figure 11 presents a comparison between the finite element simulation results and the resistance degradation model under varying activation energies and dielectric thicknesses, at a temperature of 370 K and voltage of 1 V, based on the SPICE degradation model. Figure 12 and Figure 13 illustrate the comparison of time-to-failure under rectangular and sinusoidal voltage waveforms, respectively, at different temperatures while keeping all other parameters constant. These analyses aim to evaluate the applicability and robustness of the model under various operating conditions, demonstrating its adaptability to different stress scenarios.

### 4.3. Analysis of Weibull Distribution Model Results

**Figure 14:**
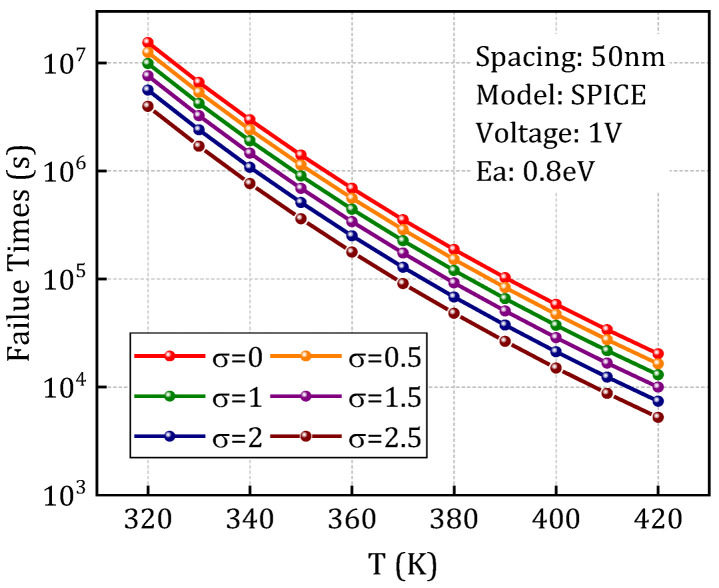
Time-to-failure at different amplitude values under varying temperatures.

Further, the process tolerance model depicted in Section 3.2 is integrated to account for the influence of inevitable process variations on the degradation behavior of dielectric layers. By leveraging the statistical distribution characteristics of the PSD function, appropriate random seeds were generated to simulate the variability induced by LER. As shown in Figure 14, the time-to-failure (TTF) was evaluated across different LER amplitude levels defined by the PSD function. The results reveal a pronounced reduction in failure time with increasing LER amplitude, indicating that LER accelerates the degradation process. The results indicate that as the LER amplitude increases, the failure time decreases significantly, demonstrating the acceleration effect of LER on the degradation rate. This highlights the nonlinear amplification of microscopic geometric perturbations on macroscopic degradation behavior.

Subsequently, 100 independent random seeds are generated to perform multiple simulation trials. The resulting 100 TTF datasets are then sorted in ascending order. To mitigate the influence of extreme values on the statistical analysis, the lowest and highest 10% of the data points are excluded as outliers. The remaining 80 datasets are utilized to fit a Weibull distribution model, enabling the extraction of key statistical parameters characterizing the degradation behavior.

**Figure 15:**
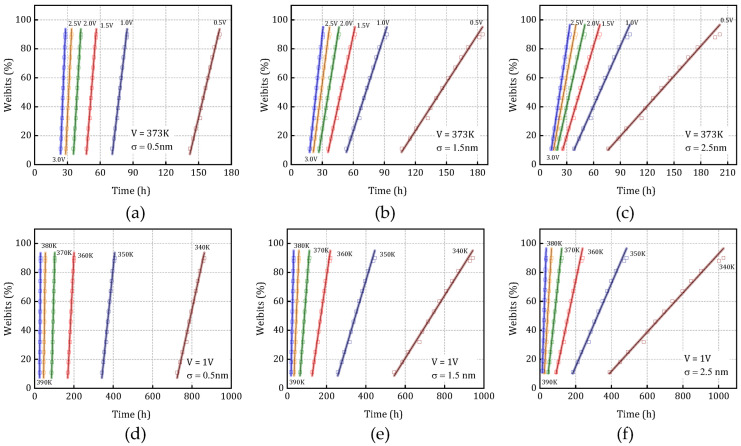
(**a**–**c**) Weibull distributions for different *σ* values under varying voltages and (**d**–**f**) Weibull distributions for different *σ* values under varying temperatures. Markers represent simulation data, and lines correspond to fitted Weibull distribution curves. The shaded areas indicate the lifetime distribution within the 95% confidence interval.

**Figure 16:**
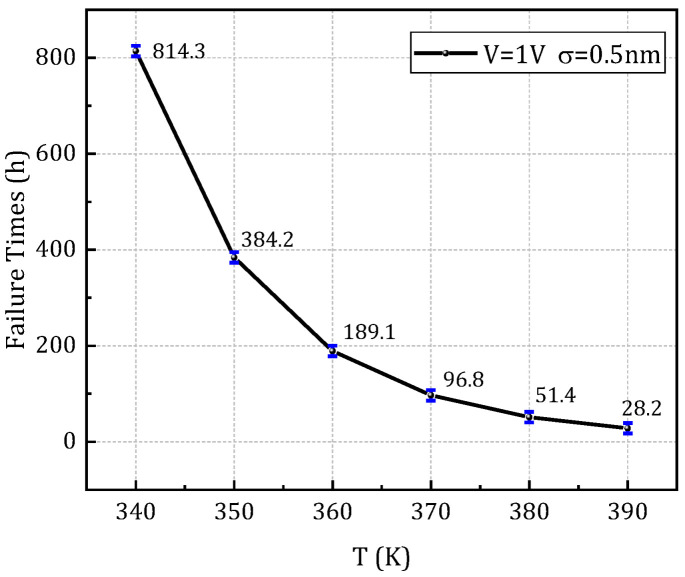
Lifetime prediction curve as a function of temperature in case of LER σ = 0.5 nm, V = 1 V.

Figure 15 illustrates the Weibull distribution characteristics corresponding to different LER (line edge roughness) amplitudes under different temperature conditions (Figure 15a–c) and voltage levels (Figure 15d–f), with a 95% confidence interval for the linear fitting. From these distributions, the characteristic failure time associated with a 63.2% cumulative failure probability-denoted as t_BD,63%_- is extracted and defined as the dielectric layer’s failure time under each stress condition. The results demonstrate that variations in LER amplitude can lead to significant shifts in the shape and scale parameters of the Weibull distribution. Furthermore, changes in temperature and voltage are shown to amplify the degradation model’s sensitivity to stochastic process variations. This highlights the pronounced impact of LER on the inherent randomness and uncertainty in the resistance degradation process. Importantly, it also reveals that environmental stressors such as temperature and voltage can markedly alter the statistical dynamics of TDDB degradation, emphasizing the necessity of incorporating both process variability and operational conditions into reliability modeling and prediction frameworks. Figure 16 shows the lifetime prediction curves under different stress conditions with an LER amplitude of 0.5 nm, and the error bars represent the statistical variability at the 95% confidence level.

## 5. Conclusions

This paper proposes a SPICE degradation simulation model for the TDDB effect in dielectric layers within devices and packages, aimed at analyzing the TDDB behavior of dielectric layers in back-end processes. The model’s accuracy under various excitation conditions and dimensional parameters was validated through finite element simulations. Subsequently, a process tolerance model incorporating the power spectral density function was introduced to quantitatively characterize LER in the dielectric layer. Combined with 100 Monte Carlo simulation runs, the impact of LER on time-to-failure was further investigated. The results demonstrate that variations in LER amplitude significantly affect the shape and scale parameters of the Weibull distribution, while different temperature and voltage conditions further amplify the degradation model’s sensitivity to stochastic perturbations. By fitting the data to a Weibull distribution and applying linear regression with a 95% confidence interval, the failure t_BD,63%_ was extracted in cases of varying voltage, temperature, and LER amplitude conditions.

## Figures and Tables

**Table 1:** Input parameter values of the EPG model [18,25].

Param	Values	Units
$E_{a}$	0.8	eV
$\varepsilon_{\mathrm{perm}}$	2.9	-
T	370	K
$D_{0}$	2.24 × 10^−11^	m^2^/s
$k_{B}$	1.38 × 10^−23^	-
$V_{DD}$	1	V

**Table 2:** Input parameters of the SPICE degradation model.

Param	Values	Units
$E_{a}$	2.86	eV
κ	1.38 × 10^−23^	J/K
a	0.2	nm
Z	1	-
q	1.6 × 10^−19^	C
χ	0.9	-
f	5 × 10^12^	Hz

**Table 3:** Relative errors between the SPICE degradation model and numerical model.

T(K)	Voltage (V)	R.E%	T(K)	Voltage (V)	R.E%
350	0.5	0.18	410	0.5	0.39
350	1.0	0.23	410	1.0	0.39
350	1.5	0.38	410	1.5	0.85
350	2.0	0.68	410	2.0	0.40
350	2.5	0.18	410	2.5	0.47
350	3.0	0.23	410	3.0	0.89
370	0.5	1.07	430	0.5	0.20
370	1.0	0.51	430	1.0	0.26
370	1.5	0.26	430	1.5	0.88
370	2.0	0.29	430	2.0	0.69
370	2.5	1.7	430	2.5	0.59
370	3.0	0.18	430	3.0	0.29
390	0.5	0.05	450	0.5	0.63
390	1.0	0.05	450	1.0	0.95
390	1.5	0.36	450	1.5	0.83
390	2.0	0.18	450	2.0	0.86
390	2.5	0.53	450	2.5	0.54
390	3.0	0.58	450	3.0	0.33

## Data Availability

Data are contained within this article.
